# Supplementary material for: Molecular patterns of resistance to immune checkpoint blockade in melanoma
Source: Nat Commun. 2024 Apr 9;15:3075. doi: 10.1038/s41467-024-47425-y (PMC11004175; doi:10.1038/s41467-024-47425-y)
Supplement: Supplementary file 1 — Supplementary Information [file 41467_2024_47425_MOESM1_ESM.pdf]

Supplementary information

Molecular patterns of resistance to immune checkpoint blockade in melanoma

Martin Lauss[1,2], Bengt Phung[1,2], Troels Borch[3], Katja Harbst[1,2], Kamila Kaminska[1,2], Anna Ebbesson[1], Ingrid Hedenfalk[1,2], Joan Yuan[4], Kari Nielsen[2,5], Christian Ingvar[6], Ana Carneiro[1,7], Karolin Isaksson[2,6,8], Kristian Pietras[2,9], Inge Marie Svane[3], Marco Donia[3,*], Göran Jönsson[1,*]

1. Division of Oncology, Department of Clinical Sciences, Faculty of Medicine, Lund University, 22185 Lund, Sweden

2. Lund University Cancer Center, LUCC, Sweden

3. National Center for Cancer Immune Therapy, Department of Oncology, Copenhagen University Hospital, Herlev, Denmark

4. Division of Molecular Hematology, Department of Laboratory Medicine, Faculty of Medicine, Lund University, 22185 Lund, Sweden

5. Division of Dermatology, Skåne University Hospital and Department of Clinical Sciences, Faculty of Medicine, Lund University, 22185 Lund, Sweden

6. Division of Surgery, Department of Clinical Sciences, Faculty of Medicine, Lund University, 22185 Lund, Sweden

7. Department of Hematology, Oncology and Radiation Physics, Skåne University Hospital Comprehensive Cancer Center, 22185 Lund, Sweden

8. Department of Surgery, Kristianstad Hospital, 29133, Kristianstad, Sweden

9. Division of Translational Cancer Research, Department of Laboratory Medicine, Faculty of Medicine, Lund University, 22185 Lund, Sweden

* These authors jointly supervised this work

Supplementary Table 1. Selected gene lists to define T cell and B cell clusters in Figure 5.

Symbol	Type
CD3D	T_clusters
CD3E	T_clusters
CD3G	T_clusters
CD247	T_clusters
LCK	T_clusters
CD8A	T_clusters
CD8B	T_clusters
IFNG	T_clusters
GZMA	T_clusters
GZMB	T_clusters
GZMK	T_clusters

PRF1	T_clusters
TNFRSF9	T_clusters
TIGIT	T_clusters
LAG3	T_clusters
PDCD1	T_clusters
HAVCR2	T_clusters
CXCL13	T_clusters
MKI67	T_clusters
CD4	T_clusters
CD40LG	T_clusters
TNF	T_clusters
ICOS	T_clusters
TNFRSF4	T_clusters
BCL6	T_clusters
CXCR5	T_clusters
LTA	T_clusters
LTB	T_clusters
IL7R	T_clusters
TCF7	T_clusters
CCR7	T_clusters
SELL	T_clusters
LEF1	T_clusters
FOXP3	T_clusters
IL2RA	T_clusters
CTLA4	T_clusters
NKG7	T_clusters
GNLY	T_clusters
KLRC1	T_clusters
KLRF1	T_clusters
CD19	B_clusters
MS4A1	B_clusters
CD79A	B_clusters
CD79B	B_clusters
TCL1A	B_clusters
IGLL1	B_clusters
CD69	B_clusters
FCER2	B_clusters
CD86	B_clusters
CD27	B_clusters
CD38	B_clusters
CR2	B_clusters
IGHD	B_clusters
IGHM	B_clusters
PAX5	B_clusters
BANK1	B_clusters
TNFRSF17	B_clusters
IRF4	B_clusters
AICDA	B_clusters
BCL6	B_clusters
MKI67	B_clusters
HLA-DRA	B_clusters
HLA-DQA1	B_clusters
CXCR5	B_clusters

LTA	B_clusters
LTB	B_clusters
CCR7	B_clusters
SELL	B_clusters
IL10	B_clusters
IGHG1	B_clusters
IGHG3	B_clusters
IGHG2	B_clusters
IGHG4	B_clusters
IGHA1	B_clusters
IGHA2	B_clusters
SDC1	B_clusters
XBP1	B_clusters
PRDM1	B_clusters
MZB1	B_clusters

Supplementary Table 2. Antibodies used in panel 1.

Sequence	Blocking (Roche, Disc antibody block)	Antibody	Manufac.	Clone	Dilution	Incubation 37 °C time (min)
1	No	PD1	Cell Marque	NAT105	RTU	32
2	No	Ki67	DAKO	MIB-1	1:100	32
3	No	CD8	Cell Marque	C8/144B	1:200	32
4	Pre-block 12 min	FOXP3	Abcam	236A/E7	1:50	32
5	No	TCF7	Invitrogen	C.725.7	1:100	60
6	No	SOX10	Biocare Medical	BC34	1:100	32

Supplementary Table 3. Antibodies used in panel 2.

Sequence	Blocking (Roche, Disc antibody block)	Antibody	Manufac.	Clone	Dilution	Incubation 37 °C time (min)
1	No	MITF	Atlas Antibodies	Polycl.	1:500	40
2	Co-block 12 min	CD20	Roche	L26	RTU	32
3	No	Ki67	DAKO	MIB-1	1:100	32
4	No	NGFR	Atlas Antibodies	Polycl.	1:200	32
5	No	CD3	Roche	2GV6	RTU	32
6	No	SOX10	Biocare Medical	BC34	1:100	32

Supplementary Table 4. Antibodies used in panel 3.

Sequence	Blocking (Roche, Disc antibody block)	Antibody	Manufac.	Clone	Dilution	Incubation 37 °C time (min)
1	Co-block 12 min	B2M	Atlas Antibodies	Polycl.	1:500	60 (RT)
2	Co-block 12 min	CD20	Roche	L26	RTU	32
3	No	MITF	Atlas Antibodies	Polycl.	1:100	40
4	No	Ki67	DAKO	MIB-1	1:100	32
5	No	CD3	Roche	2GV6	RTU	32
6	No	SOX10	Biocare Medical	BC34	1:100	32

Supplementary Table 5. TSA reagent details.

Sequence	TSA reagent (Akoya)	Dilution
1	Opal 620	1:100
2	Opal 690	1:100
3	Opal 520	1:100
4	Opal 570	1:200
5	Opal 480	1:200
6	TSA-DIG + Opal 780	1:120 + 1:30

Supplementary Figure 1

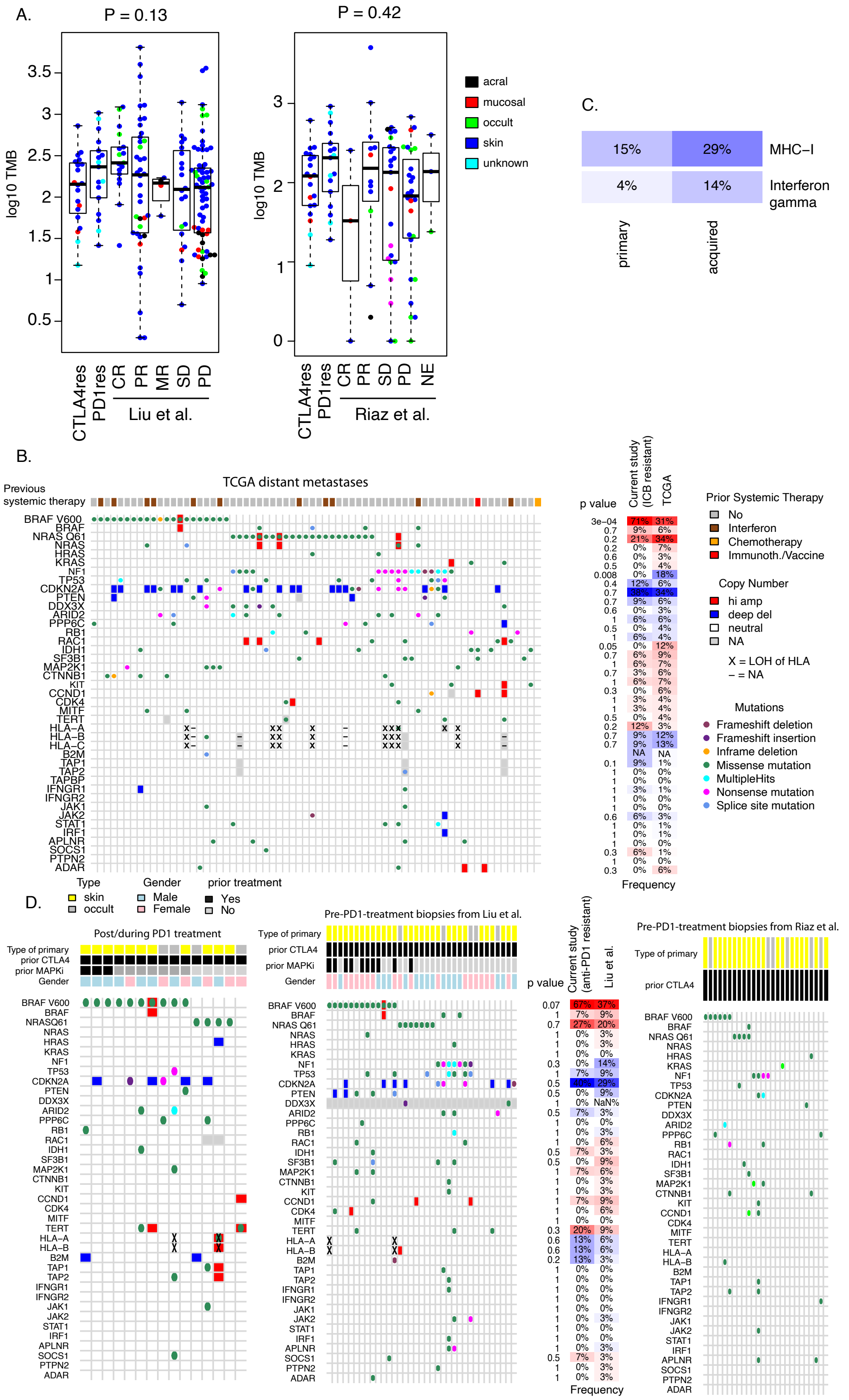

Supplementary Figure 1. Genetic alterations in public data of advanced melanoma. A. Tumor mutational burden (TMB) calculated as log-transformed total number of somatic non-silent mutations in anti-CTLA4 resistant (CTLA4res) (n=20) or anti-PD1 resistant (PD1res) (n=17) tumors as compared to data from Liu et al.[1] (n=144) and Riaz et al.[2] (n=68). The patient samples in the Riaz et al.[2] and Liu et al.[1] data were pre-treatment biopsies from patients receiving immune checkpoint blockade (ICB), while our data come from post-treatment biopsies. The RECIST response to ICB was determined for each patient in the Liu et al.[1] and Riaz et al.[2] data. CR = Complete Response, PR = Partial Response, SD = Stable Disease, PD = Progressive Disease, MR = Mixed Response, NE = Inevaluable. Boxplots are displayed with the center-line as median, the box limits as lower and upper quartiles, and with whiskers covering the most extreme values within 1.5 x Interquartile-Range. **B.** Oncomap of genetic aberrations of selected genes in distant metastases of the Cancer Genome Atlas (TCGA) cohort (n=68). Frequencies of the genetic aberrations inthe ICB resistant group (CTLA4res and PD1res) in Figure 1B and the TCGA treatment naïve group were compared, P-values from Fisher test. All tests were two-sided. **C.** Frequency plot of immune regulatory pathways between samples with primary (n=26) and acquired (n=7) resistance from CTLA4res and PD1res samples, considering only loss-of-function events. **D.** Oncomap of genetic aberrations of selected genes in PD1res samples with prior relapse to CTLA4 blockade (left, n=15), These samples were compared to two publicly available datasets, pre-PD1 treatment samples from patients that had relapsed on anti-CTLA4 from Liu et al.[1] (middle, n=35) and Riaz et al.[2] (right, n=26). Frequencies of activating events for potential oncogenes and loss-of-function events for potential tumor suppressor genes were compared between ICB resistant tumors and the Liu et al.[1] control cohort, P-values from Fisher test. All tests were two-sided. For Riaz et al[2], only mutational data were available. Source data are provided as a Source Data file.

Supplementary Figure 2

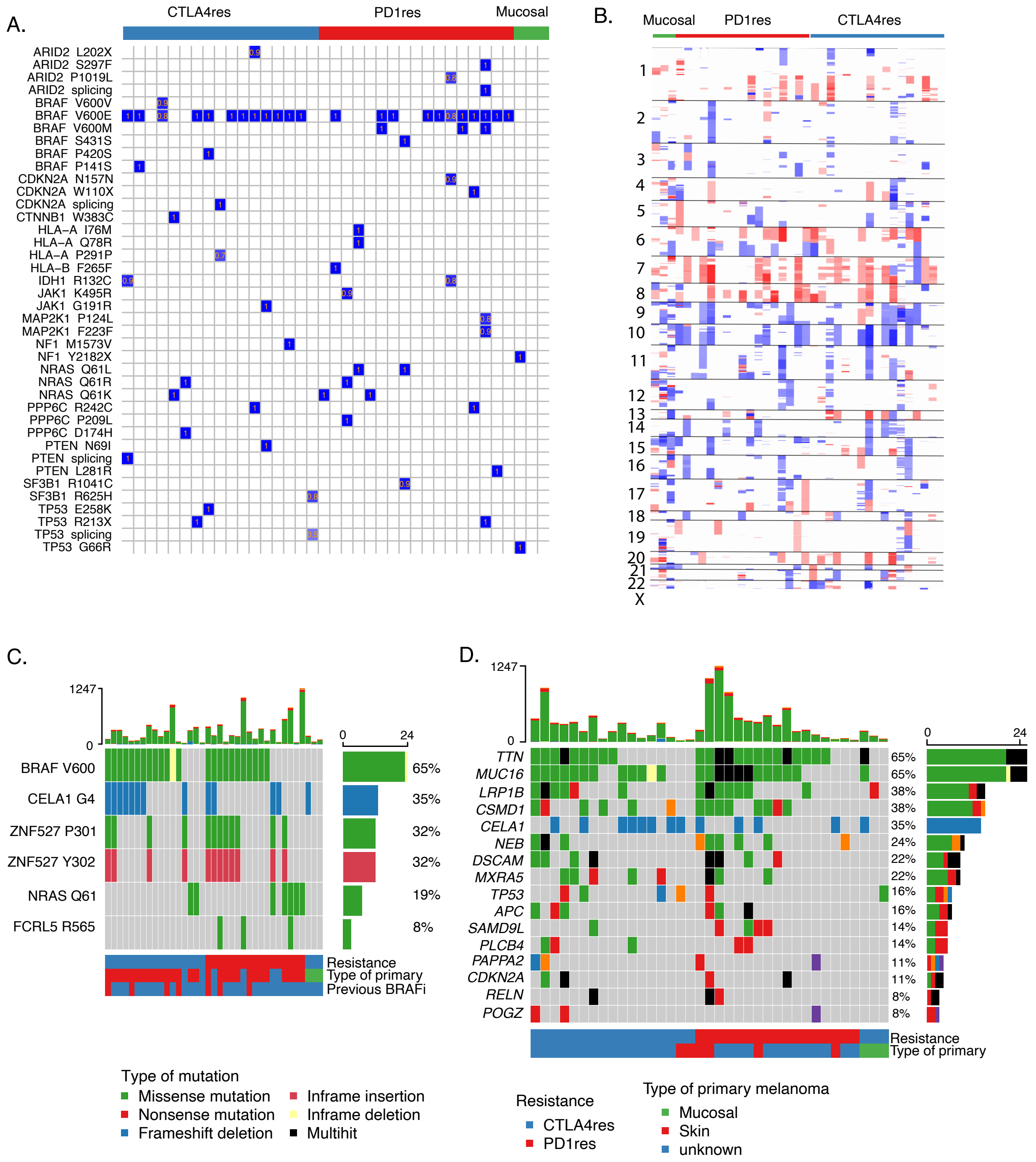

Supplementary Figure 2. Genomic architecture of immune checkpoint blockade (ICB) resistant melanoma. A. Cancer cell fraction (CCF) of melanoma key genes are plotted across all ICB resistant melanomas (17 CTLA4res, 17 PD1res and 3 mucosal). A TP53 splicing mutation was the only mutation that was called as subclonal, i.e., the CCF 95% confidence interval does not pass 0.95. CTLA4res = anti-CTLA4 resistant, PD1res = anti-PD1 resistant **B.** Genome-wide copy number profiles of ICB resistant melanoma showing similarities between the groups. Higher copy number changes were observed in the three mucosal melanomas. Blue

indicates losses and red indicates gains in relation to matched normal blood control. **C.** Oncomap of recurrent hotspot mutations, excluding silent mutations, found in at least three ICB resistant melanomas from a genome-wide analysis across 37 ICB resistant cases. **D.** Oncomap of recurrent Loss-of-Function mutations, not considering missense (green) and in-frame (yellow) events, found in at least three ICB resistant melanomas from a genome-wide analysis across 37 ICB resistant cases.

Supplementary Figure 3

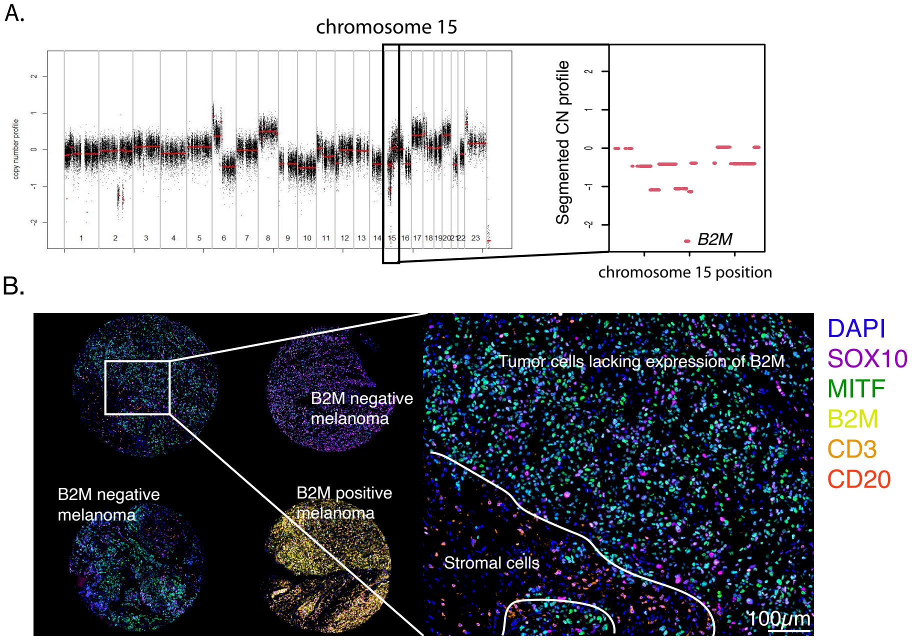

Supplementary Figure 3. B2M genetic alterations in metastatic melanoma. **A.** Genome-wide copy number profile from one case with a B2M genetic alteration generated from whole exome sequencing data demonstrates several chromosomal alterations. Zoom in of a region on chromosome 15 (+/- 8Mbp from *B2M*) shows a focal deletion of the *B2M* gene locus. **B.** Multiplex immunofluorescence analysis shows loss of B2M protein in the same tumor. As reference there are two other B2M negative and one B2M positive melanoma cores.

Supplementary Figure 4

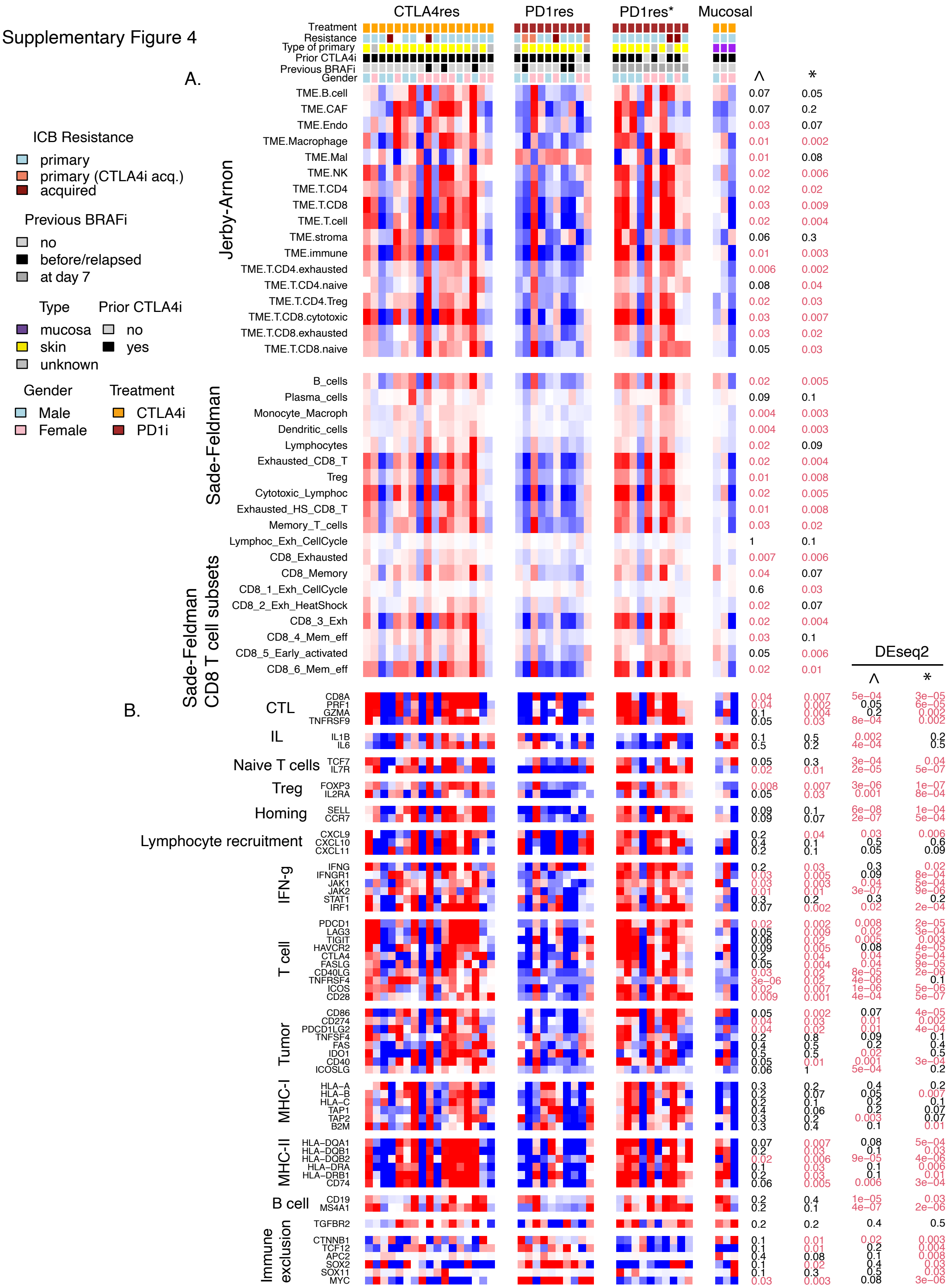

Supplementary Figure 4. Immune gene expression in bulk immune checkpoint blockade

(ICB) resistant melanoma. A. Heatmap of immune cell signatures from melanoma single cell RNA sequencing studies by Jerby-Arnon et al.[3] and Sade-Feldman et al.[4]. CTLA4res = anti-CTLA4 resistant (n=17), PD1res = anti-PD1 resistant (n=10), PD1res* = anti-PD1 resistant under BRAFi treatment (n=10). Three mucosal melanomas are included in heatmap but excluded from statistical analyses. Red = increased expression, blue = decreased expression. P-values from t-test. **B.** Heatmap of expression of single genes of relevance in key immunological processes. Grouping of samples as in A. P-values from t-test and DESeq2, as indicated. * P < 0.05 between PD1res and PD1res*. ^ P < 0.05 between PD1res and CTLA4res lesions. All tests were two-sided.

Supplementary Figure 5

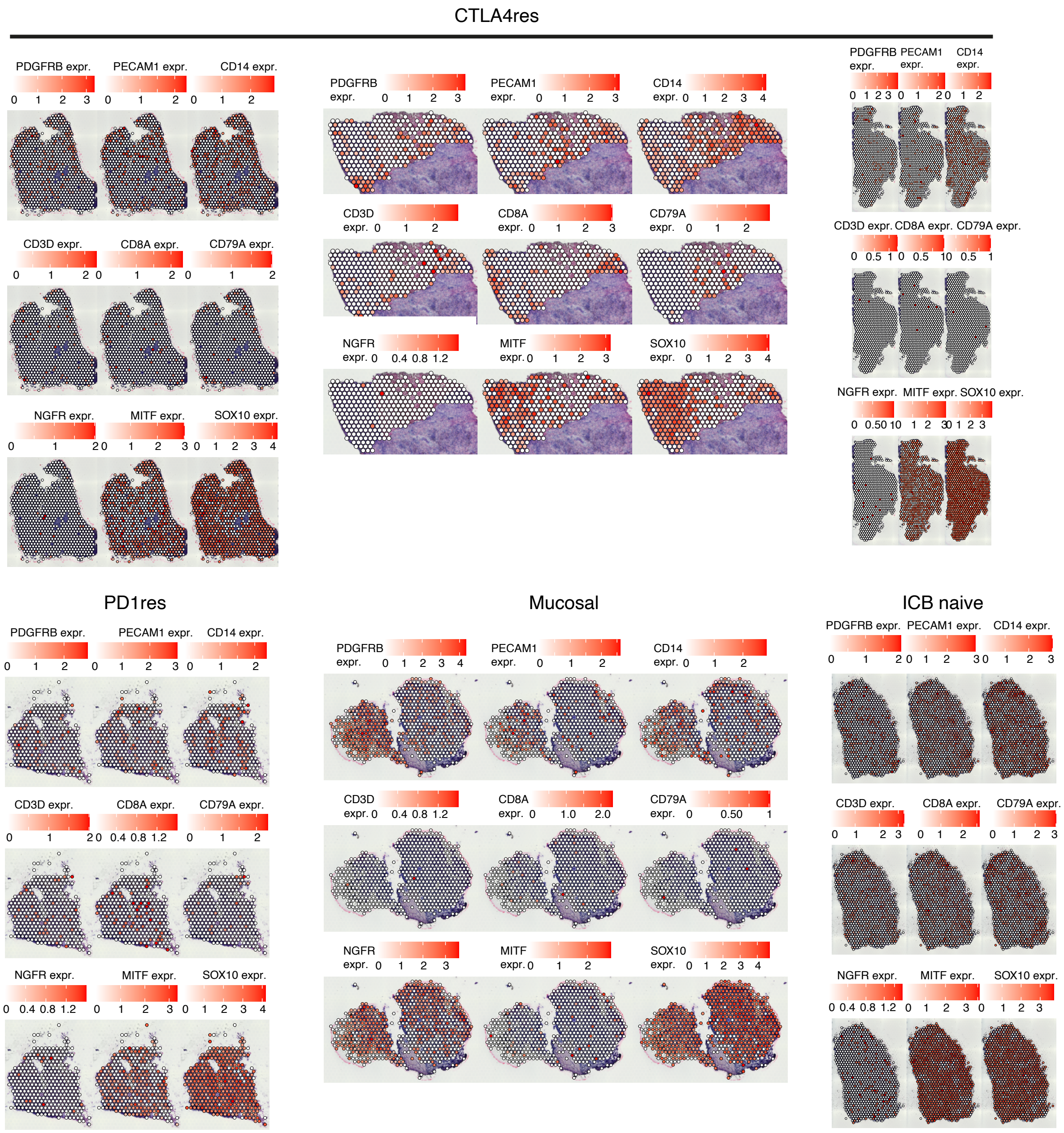

Supplementary Figure 5. Spatial gene expression of lineage marker genes. Spatial gene expression plots of all tissue sections run on the Visium platform. Expression of key lineage marker genes for cancer-associated fibroblasts (*PDGFRB*), endothelial cells (*PECAM1*), monocytes/macrophages (*CD14*), T cells (*CD3D*, *CD8*), B cells (*CD79*) and malignant cells (*NGFR*, *MITF*, *SOX10*) was plotted across the entire tissue sections. This provides an overall spatial map of different cell types present in each tissue. Data were derived for spots of 55 µm diameter, and were mapped back to histological images of six melanoma tumors (three anti-CTLA4 resistant (CTLA4res), one anti-PD1 resistant (PD1res), one immune checkpoint blockade naïve and one mucosal melanoma).

Supplementary Figure 6

A.

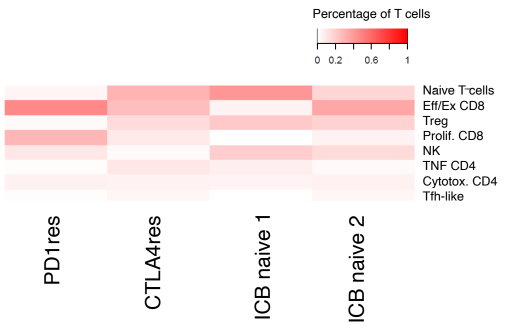

B.

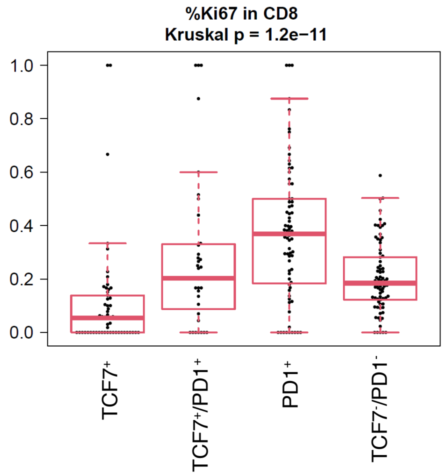

Supplementary Figure 6. T cell phenotype proportions. A. Percentages of the T cell cluster assignments using single cell RNA sequencing data of four melanoma samples with increased amount of B cells. An increased fraction of effector/exhausted $CD8^+$ T cells (Eff/Ex CD8) was observed in the anti-PD1 resistant (PD1res) melanoma. CTLA4res = anti-CTLA4 resistant, ICB naive = immune checkpoint blockade naive. Treg – regulatory T cells, Prolif. CD8 – proliferative $CD8^+$ T cells, TNF CD4 – TNF^+ $CD4^+$ T cells, Cytotox. CD4 – cytotoxic $CD4^+$ T cells, Tfh-like - T follicular helper like T cells. **B.** Fractions of $Ki67^+$ $CD8^+$ cells using combinations of $TCF7^{-/+}$ and $PD1^{-/+}$ phenotypes, derived from multiplex immunofluorescence images. P-value from Kruskal-Wallis test. $TCF7^+/PD1^-$, n=54; $TCF7^+/PD1^+$, n=35; $TCF7^-/PD1^+$, n=72; $TCF7^-/PD1^-$, n=77. Boxplot is displayed with the center-line as median, the box limits as lower and upper quartiles, and with whiskers covering the most extreme values within 1.5 x Interquartile-Range. Source data are provided as a Source Data file.

References

1 Liu, D. *et al.* Integrative molecular and clinical modeling of clinical outcomes to PD1 blockade in patients with metastatic melanoma. *Nat Med* **25**, 1916-1927 (2019). https://doi.org:10.1038/s41591-019-0654-5

2 Riaz, N. *et al.* Tumor and Microenvironment Evolution during Immunotherapy with Nivolumab. *Cell* **171**, 934-949 e915 (2017). https://doi.org:10.1016/j.cell.2017.09.028

3 Jerby-Arnon, L. *et al.* A Cancer Cell Program Promotes T Cell Exclusion and Resistance to Checkpoint Blockade. *Cell* **175**, 984-997 e924 (2018). https://doi.org:10.1016/j.cell.2018.09.006

4 Sade-Feldman, M. *et al.* Defining T Cell States Associated with Response to Checkpoint Immunotherapy in Melanoma. *Cell* **175**, 998-1013 e1020 (2018). https://doi.org:10.1016/j.cell.2018.10.038
