# Supplementary material for: Molecular patterns of resistance to immune checkpoint blockade in melanoma
Source: Nat Commun. 2024 Apr 9;15:3075. doi: 10.1038/s41467-024-47425-y (PMC11004175; doi:10.1038/s41467-024-47425-y)
Supplement: Supplementary file 3 — Description of Additional Supplementary Files [file 41467_2024_47425_MOESM3_ESM.pdf]

Description of Additional Supplementary Files

Title: Supplementary Data 1

Description: This file includes the somatically acquired mutations that were called in all cases related to figure 1b.
