# Supplementary material for: Molecular patterns of resistance to immune checkpoint blockade in melanoma
Source: Nat Commun. 2024 Apr 9;15:3075. doi: 10.1038/s41467-024-47425-y (PMC11004175; doi:10.1038/s41467-024-47425-y)
Supplement: Supplementary file 5 — Reporting Summary [file 41467_2024_47425_MOESM5_ESM.pdf]

nature portfolio

Corresponding author(s): NCOMMS-23-34421C, Göran Jönsson

Last updated by author(s): Mar 26, 2024

# Reporting Summary

Nature Portfolio wishes to improve the reproducibility of the work that we publish. This form provides structure for consistency and transparency in reporting. For further information on Nature Portfolio policies, see our Editorial Policies and the Editorial Policy Checklist.

## Statistics

For all statistical analyses, confirm that the following items are present in the figure legend, table legend, main text, or Methods section.

| n/a | Confirmed | |
|---|---|---|
| ☐ | ☒ | The exact sample size (*n*) for each experimental group/condition, given as a discrete number and unit of measurement |
| ☐ | ☒ | A statement on whether measurements were taken from distinct samples or whether the same sample was measured repeatedly |
| ☐ | ☒ | The statistical test(s) used AND whether they are one- or two-sided<br>*Only common tests should be described solely by name; describe more complex techniques in the Methods section.* |
| ☐ | ☒ | A description of all covariates tested |
| ☐ | ☒ | A description of any assumptions or corrections, such as tests of normality and adjustment for multiple comparisons |
| ☐ | ☒ | A full description of the statistical parameters including central tendency (e.g. means) or other basic estimates (e.g. regression coefficient) AND variation (e.g. standard deviation) or associated estimates of uncertainty (e.g. confidence intervals) |
| ☐ | ☒ | For null hypothesis testing, the test statistic (e.g. *F*, *t*, *r*) with confidence intervals, effect sizes, degrees of freedom and *P* value noted<br>*Give P values as exact values whenever suitable.* |
| ☒ | ☐ | For Bayesian analysis, information on the choice of priors and Markov chain Monte Carlo settings |
| ☐ | ☒ | For hierarchical and complex designs, identification of the appropriate level for tests and full reporting of outcomes |
| ☐ | ☒ | Estimates of effect sizes (e.g. Cohen's *d*, Pearson's *r*), indicating how they were calculated |

*Our web collection on statistics for biologists contains articles on many of the points above.*

## Software and code

Policy information about availability of computer code

| | |
|---|---|
| Data collection | Data were collected using R version 4.0.5. |
| Data analysis | Bioinformatical analyses were performed using R version 4.0.5 and the packages limma 2.44.3, swamp 1.5.1, beeswarm 0.3.1, gplots 3.0.1.1, clusterProfiler 3.16.1, DESeq2 1.28.1, ConsensusClusterPlus 1.52.0, maftools 2.4.12, deconstructSigs, GLAD, ABSOLUTE, sctransform 0.3.2 and Seurat 4.0.1. Further, we used the softwares MiXCR, VDJtools, HISAT2 2.1.0, StringTie, SpaceRanger, samtools, bcftools, CONTRA 2.0.3, SAREK pipeline, VarScan 2.4.2 and Strelka2. For image analyses, InForm 2.4.11 and QuPath 0.3.2 were used. Computational code is available from the corresponding author on reasonable request. |

For manuscripts utilizing custom algorithms or software that are central to the research but not yet described in published literature, software must be made available to editors and reviewers. We strongly encourage code deposition in a community repository (e.g. GitHub). See the Nature Portfolio guidelines for submitting code & software for further information.

## Data

Policy information about availability of data

All manuscripts must include a data availability statement. This statement should provide the following information, where applicable:

- Accession codes, unique identifiers, or web links for publicly available datasets
- A description of any restrictions on data availability
- For clinical datasets or third party data, please ensure that the statement adheres to our policy

Public mutational data was downloaded from TCGA Pan-Cancer Atlas (gdc.cancer.gov/about-data/publications/pancanatlas), Liu et al.38 and Riaz et al.39. Gene

signatures were obtained from referenced publications, respectively. Publicly available data with accession numbers GSE115978 [https://www.ncbi.nlm.nih.gov/geo/query/acc.cgi?acc=GSE115978] and GSE120575 [https://www.ncbi.nlm.nih.gov/geo/query/acc.cgi?acc=GSE120575] were downloaded from Gene Expression Omnibus (GEO) and were used to identify tumor-specific genes.
Processed bulk RNA sequencing and single cell RNA sequencing data have been deposited at GEO with accession number GSE244982 [https://www.ncbi.nlm.nih.gov/geo/query/acc.cgi?acc=GSE244982] and GSE244983 [https://www.ncbi.nlm.nih.gov/geo/query/acc.cgi?acc=GSE244983]. Raw data are not available for these GEO submissions, as due to Swedish and Danish laws, the patient consent, and the risk that the sequencing data contains personally-identifiable information and hereditary mutations, we cannot deposit the short sequencing read data in a public access repository.
Spatial transcriptomics data have been deposited under accession number GSE261347 [https://www.ncbi.nlm.nih.gov/geo/query/acc.cgi?acc=GSE261347].
Whole exome sequencing- and T cell receptor sequencing data were deposited in European Genome Archive (EGA) under EGAD50000000380 and EGAD50000000379, respectively. These data are available under restricted access. Data access can be granted via the EGA under collaborative conditions and when aligned with current ethical approval, and data will be available for duration of the proposed project. Somatically called mutations are available as Supplementary Data 1.
All other remaining data are available within the Article, Supplementary Information or as Source data file. Source data are provided with this paper.

## Research involving human participants, their data, or biological material

Policy information about studies with human participants or human data. See also policy information about sex, gender (identity/presentation), and sexual orientation and race, ethnicity and racism.

| | |
|---|---|
| Reporting on sex and gender | Metastatic melanoma affects both male and females. Sex was collected from clinical chart records but was not included as part of any analyses. |
| Reporting on race, ethnicity, or other socially relevant groupings | Non applicable |
| Population characteristics | Patient characteristics were collected from clinical chart records and are presented in Table 1. |
| Recruitment | Metastatic melanoma tissue were collected from patients that had progressed on ICB and been enrolled in TIL therapy trials. ICB naive patients included as controls were collected at Skåne University Hospital before clinical introduction of ICB in Sweden. |
| Ethics oversight | All three trials (NCT00937625, NCT02354690, NCT02379195) are listed in clinicaltrials.gov, and all procedures were conducted in accordance with the Declaration of Helsinki and following approval from the Scientific Ethics Committee of the Capital Region of Denmark. Metastatic melanoma lesions from ICB naïve patients were collected at Skåne University Hospital in Sweden prior clinical introduction of immune checkpoint blockade under the ethical permit Dnr. 101/2013 and 191/2007. |

Note that full information on the approval of the study protocol must also be provided in the manuscript.

# Field-specific reporting

Please select the one below that is the best fit for your research. If you are not sure, read the appropriate sections before making your selection.

[x] Life sciences  [ ] Behavioural & social sciences  [ ] Ecological, evolutionary & environmental sciences

For a reference copy of the document with all sections, see nature.com/documents/nr-reporting-summary-flat.pdf

# Life sciences study design

All studies must disclose on these points even when the disclosure is negative.

| | |
|---|---|
| Sample size | The ICB resistant samples contain 23 metastases from patients developing resistance to anti-CTLA4 monotherapy and 21 metastases from patients developing resistance to anti-PD1 monotherapy. This is to date one of the largest cohorts of ICB-progressed patients and has been collected over the course of three clinical trials. In addition 53 ICB naive tumors were included as controls for mIF stainings. For single cell RNA sequencing, a sufficiently large amount of B and T cells to obtain stable subgroups was sequenced from four representative samples. For spatial RNA sequencing, a sufficiently large amount of spatially aligned spots to obtain tumor cell phenotypes were sequenced from six representative samples. |
| Data exclusions | For whole exome sequencing, samples with high content of non-tumor cells, resulting in scarce mutation calls, low variant allele frequencies and non-aberrated copy number profiles, were excluded. For RNA sequencing, samples with too little mRNA starting material were excluded. For mIF analyses, tissue microarray cores with absent or insufficient tumor content where excluded. |
| Replication | A single melanoma tumor was assessed for each patient. Findings on the transcriptomic level were validated on the protein level. |
| Randomization | Randomization is not relevant for this study |
| Blinding | Blinding is not relevant for this study |

# Reporting for specific materials, systems and methods

We require information from authors about some types of materials, experimental systems and methods used in many studies. Here, indicate whether each material, system or method listed is relevant to your study. If you are not sure if a list item applies to your research, read the appropriate section before selecting a response.

## Materials & experimental systems

| n/a | Involved in the study |
| --- | --- |
| ☐ | ☒ Antibodies |
| ☒ | ☐ Eukaryotic cell lines |
| ☒ | ☐ Palaeontology and archaeology |
| ☒ | ☐ Animals and other organisms |
| ☒ | ☐ Clinical data |
| ☒ | ☐ Dual use research of concern |
| ☒ | ☐ Plants |

## Methods

| n/a | Involved in the study |
| --- | --- |
| ☒ | ☐ ChIP-seq |
| ☒ | ☐ Flow cytometry |
| ☒ | ☐ MRI-based neuroimaging |

# Antibodies

Antibodies used

PD1, Cell Marque, cat.nr. 315M-98, clone NAT105, lot V0002317
Ki67, Agilent DAKO, cat.nr. GA62661-2, clone MIB-1, lot 20079180
CD8, Cell Marque, cat.nr. 108M-96, clone C8/144B, lot 0000092372
TCF7, Invitrogen, cat.nr. MA5-14965, clone C.725.7, lot YA3795811
SOX10, Biocare Medical, cat.nr. 3099, clone BC34, lot 111919
MITF, Atlas Antibodies, cat.nr. HPA003259, Polyclonal, lot B114205
CD20, Roche, cat.nr. 05267099001, clone L26, lot H33751
NGFR, Atlas Antibodies, cat.nr. HPA004765, Polyclonal, lot GR3238403-7
CD3, Roche, cat.nr. 05278422001, clone 2GV6, lot G32274
B2M, Atlas Antibodies, cat.nr. HPA006361, Polyclonal, lot 000005424

Validation

Optimization of multiplex immuno-fluorescence involved individual singleplex testing of each antibody using chromogenic DAB staining. To assess epitope stability, the antibody of interest was placed in the staining sequence for serial sections of tonsil and melanoma tissue samples at positions 1, 3, and 5. Visual assessment of antibody sensitivity and specificity was performed to determine the optimal sequence placement within the multiplex panel. Furthermore, to enhance the sensitivity, specificity, and minimize potential cross-reactivity between antibodies in the multiplex immuno-fluorescence panel, a systematic optimization approach was employed. Each antibody was placed in different staining sequences, and their performance was evaluated to fine-tune the optimal arrangement for the multiplex panel.
